# Efficacy of the hypoxia-activated prodrug evofosfamide (TH-302) in nasopharyngeal carcinoma in vitro and in vivo

**DOI:** 10.1186/s40880-018-0285-0

**Published:** 2018-05-03

**Authors:** Yan Huang, Ying Tian, Yuanyuan Zhao, Cong Xue, Jianhua Zhan, Lin Liu, Xiaobo He, Li Zhang

**Affiliations:** 1 State Key Laboratory of Oncology in South China, Collaborative Innovation Center for Cancer Medicine, Guangdong Key Laboratory of Nasopharyngeal Carcinoma Diagnosis and Therapy, Sun Yat-sen University Cancer Center, Guangzhou, 510060 Guangdong P. R. China; 2 Department of Medical Oncology, Sun Yat-sen University Cancer Center, Guangzhou, 510060 Guangdong P. R. China; 3 Department of Experimental Research, Sun Yat-Sen University Cancer Center, Guangzhou, 510060 Guangdong P. R. China; 4 Department of Medical Oncology, The Fifth Affiliated Hospital, Sun Yat-Sen University, Zhuhai, 519000 Guangdong P. R. China; 5 Department of Radiation Oncology, The Fifth Affiliated Hospital, Sun Yat-Sen University, Zhuhai, 519000 Guangdong P. R. China

**Keywords:** Nasopharyngeal carcinoma (NPC), Hypoxia-induced factor-1α (HIF-1α), Hypoxia-activated prodrug, Chemotherapy, Xenograft tumor models

## Abstract

**Background:**

Tumor hypoxia is considered an important factor in metastasis and disease relapse. Evofosfamide is a hypoxia-activated prodrug that selectively targets the hypoxic regions of solid tumors. As hypoxia-inducible factor-1α (HIF-1α) is overexpressed in nasopharyngeal carcinoma (NPC) tissues, we performed the present study to evaluate the efficacy profile of evofosfamide in NPC.

**Methods:**

We evaluated the efficacy of evofosfamide as a single agent or combined with cisplatin (DDP) in the NPC cell lines CNE-2, HONE-1 and HNE-1, and in nude mouse xenograft tumor models.

**Results:**

Evofosfamide exhibited hypoxia-selective cytotoxicity in NPC cell lines, with 50% inhibition concentration (IC_50_) values of 8.33 ± 0.75, 7.62 ± 0.67, and 0.31 ± 0.07 μmol/L under hypoxia in CNE-2, HONE-1 and HNE-1 cells, respectively. The sensitization ranged from ninefold to greater than 300-fold under hypoxia compared with normoxia controls. The combination of evofosfamide with DDP had a synergistic effect on cytotoxicity in the NPC cell lines by combination index values assessment. Cell cycle G2 phase was arrested after treated with 0.05 μmol/L evofosfamide under hypoxia. Histone H2AX phosphorylation (γH2AX) (a marker of DNA damage) expression increased while HIF-1α expression suppressed after evofosfamide treatment under hypoxic conditions. In the HNE-1 NPC xenograft models, evofosfamide exhibited antitumor activity both as a single agent and combined with DDP. Hypoxic regions in xenograft tissue were reduced after both evofosfamide monotherapy and combined therapy with DDP.

**Conclusions:**

Our results present preclinical evidence for targeting the selective hypoxic portion of NPC by evofosfamide as a single agent and combined with DDP and provide rationale for the potential clinical application of evofosfamide for the treatment of nasopharyngeal carcinoma.

## Background

Hypoxic regions are a common feature of many solid tumors [1, 2]. Tumor hypoxia is associated with resistance to chemotherapy and radiotherapy [3]. As hypoxic tumor cells are considered to be more aggressive, invasive, and metastatic than normoxic cells, tumor hypoxia is an important factor in tumor treatment failure, recurrence, and metastasis [4].

Nasopharyngeal carcinoma (NPC) occurs specifically and commonly in Southeast Asia and South China and is a cause of very serious health problems in these areas [5]. Even with combined radiation and chemotherapy treatment the prognosis of advanced NPC is not ideal, with disease relapse rates as high as 82% [6]. Previous studies [7, 8] have shown that hypoxia-induced factor-1α (HIF-1α) is overexpressed in NPC tissues compared with normal nasopharyngeal epithelial tissues. Overexpression of HIF-1α is significantly correlated with TNM stage, lymph node metastasis and distant metastasis, and a poor prognosis. Therefore, hypoxic tumor cells are regarded as an important factor for metastasis and relapse of NPC and there is an urgent need for novel approaches that target the hypoxic regions of NPC.

Hypoxia-activated prodrugs can selectively target hypoxic tumor cells. Evofosfamide (TH-302) is a 2-nitroimidazole triggered bromo-isophosphoramide mustard with hypoxic selective cytotoxicity [9]. Evofosfamide exhibits broad antitumor activity in preclinical models of soft tissue sarcoma [10], pancreatic cancer [11], multiple myeloma [12], and non-small cell lung cancer (NSCLC) [13, 14]. Evofosfamide has also been investigated in phase II clinical trials for soft tissue sarcoma [15] and pancreatic cancer [16] with promising results.

Although several chemical agents, including targeted therapies, have been tested for the treatment of NPC in recent years, the survival of patients with advanced disease has not improved much and no standard regimens have been acknowledged [17]. Hypoxic regions are a common feature of many solid tumors, and HIF-1α was shown to be overexpressed in NPC tissues compared with normal nasopharyngeal epithelial tissues. In the present study we studied the antitumor activity of the selective hypoxia-activated prodrug evofosfamide in preclinical models of NPC. As platinum-based therapy is the preferred regimen for the therapeutic management of NPC [18], we also evaluated the efficacy of evofosfamide combined with cisplatin.

## Materials and methods

### Cell lines and culture conditions

Three poorly differentiated human NPC cell lines CNE-2, HONE-1, and HNE-1, were maintained in RPMI 1640 medium supplemented with 10% fetal bovine serum (Gibco Invitrogen, CA), penicillin (100 units/ml), and streptomycin (100 units/ml) at 37 °C in a humidified 5% CO_2_ air atmosphere (normoxic condition) or in a humidified, 5% CO_2_, 0.1% O_2_ sealed chamber (hypoxic condition). Logarithmically growing cells were used in all experiments.

### Drugs and reagents

Evofosfamide was provided by Merck KGaA (Darmstadt, Germany). For in vitro studies, evofosfamide was dissolved in dimethyl sulfoxide (DMSO) to a stock concentration of 100 mmol/L and stored at − 20 °C. The stock was diluted in fresh culture medium immediately before use and the final concentration of DMSO never exceeded 0.1%. Evofosfamide was dissolved in sterile phosphate buffered saline (PBS) for in vivo studies. Cisplatin (DDP; Hospira Australia Pty Ltd, Victoria, Australia) was obtained as a commercial product from our hospital pharmacy. Cell counting kit-8 (CCK-8) was purchased from Dojindo (Tokyo, Japan). The antibody against HIF-1α was purchased from Becton–Dickinson and Company (Franklin, NJ, USA). Antibody against phospho-histone H2AX (Ser139), Alexa Fluor 488-conjugated antibody against phospho-histone H2AX (Ser139) and glyceraldehyde-3-phosphate dehydrogenase (GAPDH) were purchased from Cell Signaling Technology (Danvers, MA, USA). Pimonidazole and anti-pimonidazole antibodies were from HPI, Inc. (Burlington, MA, USA).

### Cell viability assay

Cell viability was assessed by the CCK-8 assay according to the manufacturer’s instructions [19, 20]. Cells were seeded in 96-well plates and allowed to attach for 24 h. Evofosfamide was added at graded concentrations (0.78, 1.56, 3.125, 6.25, 12.5, 25, 50, 100 μmol/L) and the cells were incubated under the indicated hypoxic or normoxic conditions for 24 h. After removal of the drug the cells were cultured in complete medium under normoxic conditions for another 24 h. For sequential combination treatment, the cells were incubated with DDP under hypoxic or normoxic conditions for a further 48 h. Dose response curves and the 50% inhibition concentration (IC_50_) were calculated. The hypoxia cytotoxicity ratio (HCR) was calculated as IC_50_ under normoxia versus IC_50_ under hypoxia. Drug synergy was determined by the combination index (CI), which was calculated using Calcusyn software (Biosoft, Cambridge, UK) [21]. A CI of 1 indicates an additive effect between two agents, whereas a CI < 1 or > 1 indicates synergism or antagonism, respectively. All experiments were performed in triplicate in two or more independent experiments.

### Clone formation assay

Cells were seeded in 6-well plates 24 h before drug treatment. Cells were incubated with the indicated concentration (0.01, 0.1, 1, 10, 100 μmol/L) of evofosfamide for 6 h under hypoxic or normoxic conditions. The drug was removed by replacing the medium with fresh complete medium and the cells were cultured under normoxic conditions for 7–10 day. Colonies were fixed and stained with crystal violet. Clonal colonies that contained more than 50 cells were counted and the surviving fraction was calculated by dividing the clonal efficiency of treated cells by that of untreated cells.

### Flow cytometry

Cells were seeded in plates 24 h before drug treatment and then treated with the indicated concentrations of evofosfamide under hypoxic or normoxic conditions for 24 h. For cell cycle and apoptosis analysis, the cells were fixed in 70% ethanol and stored at − 20 °C overnight. The cells were stained with propidium iodide (PI) with protection from light at room temperature for 30 min and were detected using flow cytometry (Cytomics™ FC 500, Beckman Coulter, Inc., Brea, CA, USA). The DNA content was analyzed using CELL Quest software (Becton, Dickinson, and Company, Franklin Lakes, NJ, USA). Apoptosis was assessed by sub-G1 phase analysis.

H2AX is required for checkpoint-mediated cell cycle arrest and DNA repair following induction of DNA double-strand breaks. DNA damage results in the rapid phosphorylation of H2AX at Ser139 [22]. To detect phospho-histone H2AX (γH2AX), cells were permeabilized with methanol, incubated with Alexa Fluor 488-conjugated γH2AX monoclonal antibody for 2 h, and analyzed by flow cytometry (Cytomics™ Gallios, Beckman Coulter, Inc.).

### Western blotting

Cells were harvested and lysed in cell lysis buffer (Cell Signaling Technology, MA). The proteins were resolved by sodium dodecyl sulfate–polyacrylamide gel electrophoresis (SDS-PAGE) and transferred onto polyvinylidene fluoride (PVDF) membranes (Roche, Basel, Switzerland). The membranes were incubated with primary antibodies against HIF-1α, phospho-histone H2AX (Ser139), and GAPDH overnight at 4 °C. After incubation with HRP-conjugated secondary antibody for 1 h at room temperature, bands were detected using an enhanced chemiluminescence (ECL) system (Cell Signaling Technology). GAPDH served as an internal reference.

### Xenograft models and antitumor activity in vivo

All animal experiments were conducted in accordance with the Guidelines for the Welfare of Animals in Experimental Neoplasia [23]. Male BALB/c nude mice aged 6–8 weeks were supplied by Guangdong Medical Laboratory Animal Center (Guangzhou, Guangdong, China). HNE-1 cells (2 × 10^6^ cells in PBS) were injected subcutaneously into the right flanks of nude mice. The body weight of the mice and tumor size were measured and recorded twice a week. The tumor volume was calculated by the following formula: Volume (mm^3^) = length × width^2^ × 0.5. When the mean tumor volume reached approximately 100–200 mm^3^, the mice were randomly assigned by the random number table method into six groups (*n *= 10–11/group) with approximately equivalent ranges of tumor volume between groups. Evofosfamide (50 or 75 mg/kg) was administered by intraperitoneal injection twice a week as a single agent or in combination. DDP (3 mg/kg) was administered by intraperitoneal injection once a week as a single agent or in combination. Intraperitoneal injection of 0.9% NaCl was administered to the controls. All groups were treated for 2 weeks. Antitumor activity was assessed by tumor growth inhibition (TGI; the ratio of the change in mean tumor volume of the treated group to that of the control group) and tumor growth delay (TGD; TGD_500_ and TGD_1000_ were determined as the average increase in time for the treated tumor to reach a size of 500 or 1000 mm^3^ compared with that of the control group). The mice were sacrificed 21 day after treatment and tumor tissues were harvested. The harvested tumor specimens were weighed, fixed in 10% buffered formalin, and embedded in paraffin.

### Immunohistochemistry

Pimonidazole was intraperitoneally injected at 60 mg/kg body weight 1 h before animals were sacrificed. The tumor tissues were collected immediately after sacrifice and fixed in 4% paraformaldehyde and embedded in paraffin. Hematoxylin and eosin (H&E) staining was performed to assess tumor morphology. Immunohistochemical staining was performed on formalin-fixed, paraffin-embedded tumor tissue sections. The standard avidin–biotin complex–peroxidase method was used for pimonidazole staining. Slides were observed using a Nikon eclipse 80i microscope at 40× or 100× magnification. Pimonidazole-positive regions were extracted using Image-Pro Plus 6.0 (Media Cybernetics).

### Statistical analysis

Statistical analysis was performed using SPSS version 16.0 software (SSPS, Chicago, IL, USA). The data were expressed as mean values ± standard deviation. Differences in the mean values were assessed using one-way analysis of variance. Two-sided *P *< 0.05 was considered statistically significant.

## Results

### Evofosfamide exhibits hypoxia-selective cytotoxicity and synergistic efficacy with DDP in NPC cell lines

**Fig. 1:**
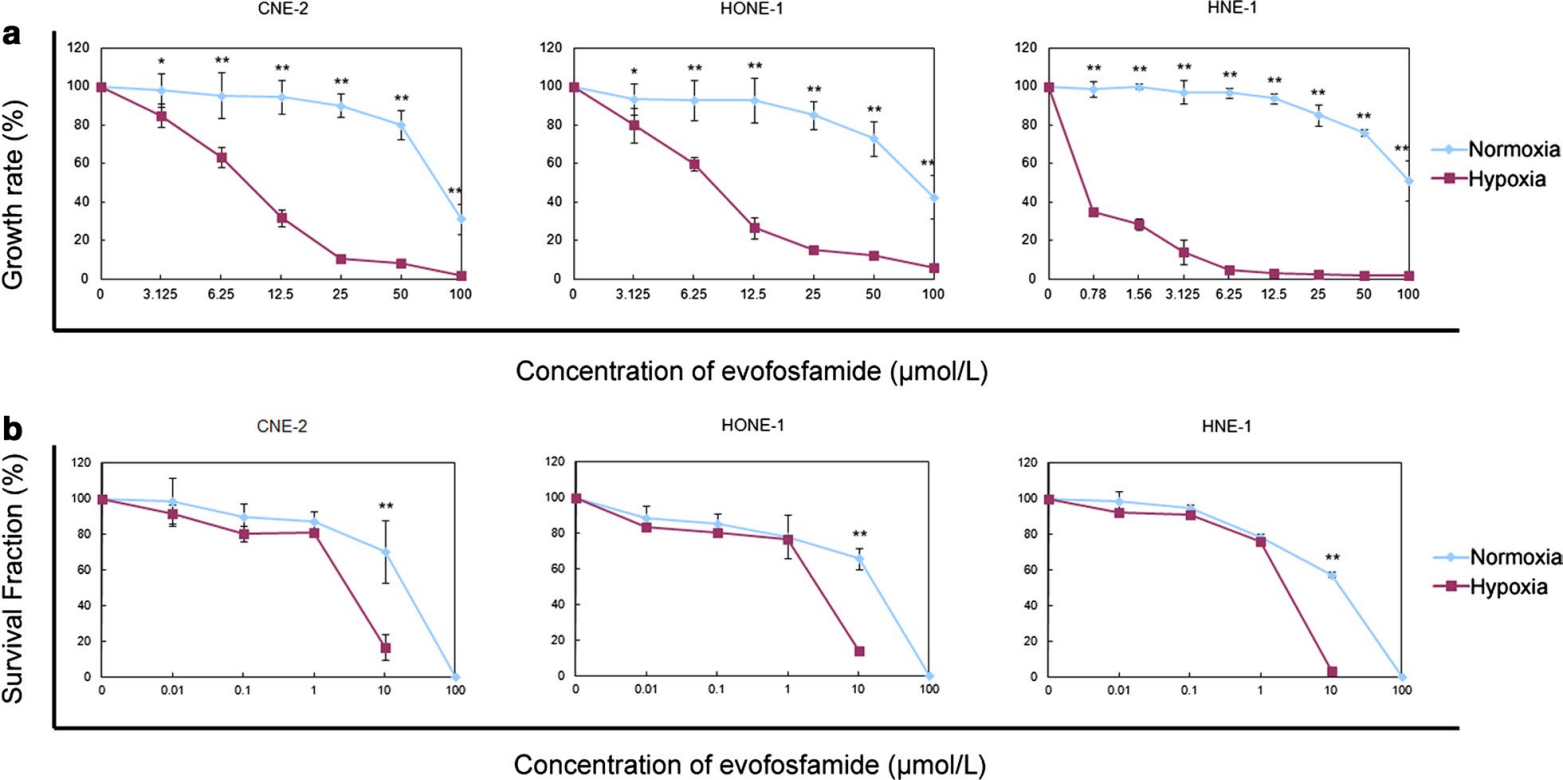
Cell growth curve and clonogenic curve of nasopharyngeal carcinoma (NPC) cell lines after evofosfamide treatment. **a** Cells were treated with evofosfamide for 24 h under hypoxia or normoxia and then cultured without drug for up to 48 h. Cytotoxicity was assessed by the cell counting kit-8 (CCK-8) assay, and the 50% inhibition concentration (IC_50_) values were calculated. The IC_50_ values and hypoxia cytotoxicity ratio (HCR) (normoxia versus hypoxia) are shown in Table 1. **b** Cells were treated with evofosfamide for 6 h under hypoxia or normoxia and then cultured without drug for 7–10 day. The clones were fixed and stained with crystal violet. Colonies of more than 50 cells were counted and the survival fractions were calculated. *P < 0.05, **P < 0.01

The NPC cell lines CNE-1, HONE-1, and HNE-1 were treated with increasing concentrations of evofosfamide. As shown in Fig. 1a and Table 1, evofosfamide exhibited modest cytotoxicity under normoxia with all IC_50_ values greater than 70 μmol/L and greater cytotoxicity under hypoxia with all IC_50_ values less than 10 μmol/L. Selectivity for hypoxia was remarkable in HNE-1 cells with HCR (IC_50_ under normoxia vs. IC_50_ under hypoxia) greater than 300-fold. Cell clonality was significantly reduced at concentrations of 10 μmol/L under hypoxia compared with 100 μmol/L under normoxia, confirming the hypoxia-selective cytotoxicity of evofosfamide (Fig. 1b).

**Table 1:** Summary of the 50% inhibition concentration (IC50) of evofosfamide in nasopharyngeal carcinoma cell lines

Cell line	IC_50_ of evofosfamide		HCR (N/H)
	N (μmol/L)	H (μmol/L)	
CNE-2	77.62 ± 8.86	8.33 ± 0.75**	9
HONE-1	87.18 ± 19.19	7.62 ± 0.67**	11
HNE-1	103.97 ± 12.91	0.31 ± 0.07**	335

*N* normoxia, *H* hypoxia, *HCR* hypoxia cytotoxicity ratio

**P < 0.01

For the combination treatment, most of the CI values were less than 1. Our results indicated that evofosfamide combined with DDP acted synergistically in the HNE-1 cell line. However, 100-times the concentration of evofosfamide under normoxia can only get an almost equal effect as it under hypoxia (Table 2).

**Table 2:** Combination index values of evofosfamide combined with cisplatin (DDP) in the HNE-1 cell line

Normoxia			Hypoxia		
Drug concentration (μmol/L)		CI	Drug concentration (μmol/L)		CI
DDP	Evofosfamide		DDP	Evofosfamide	
0.62	6.2	0.899	0.62	0.06	0.737
1.25	12.5	1.077	1.25	0.13	0.915
2.5	25	0.885	2.5	0.25	0.840
5	50	0.430	5	0.5	0.556
10	100	0.182	10	1	0.442

CI > 1 indicates an antagonistic effect; CI = 1 indicates an additive effect; CI < 1 indicates a synergistic effect

*CI* combination index

### Evofosfamide induces cell cycle arrest at G2 phase without apoptosis

**Fig. 2:**
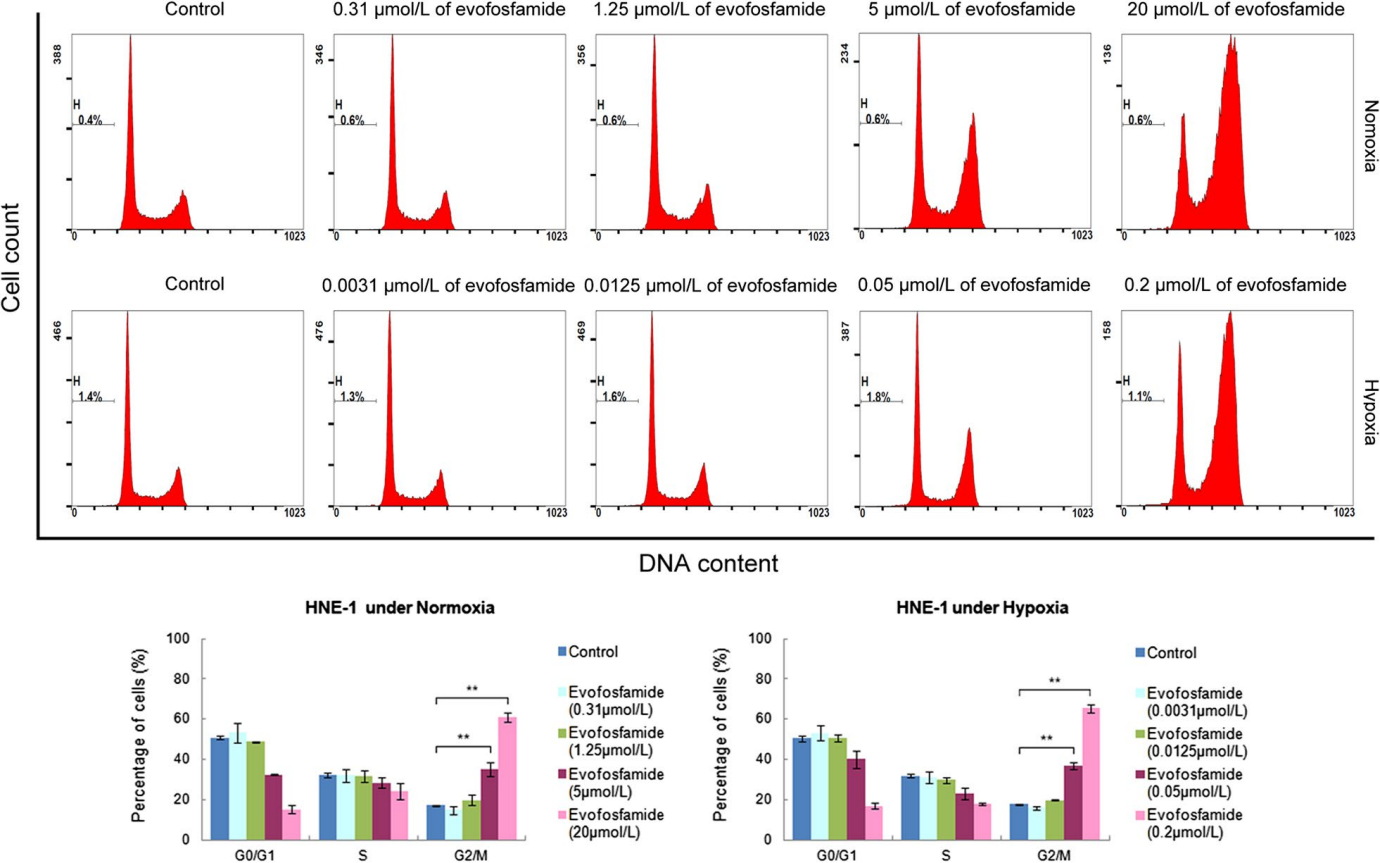
Analysis of cell cycle distribution by flow cytometry. HNE-1 cell were treated with evofosfamide for 24 h and the cell cycle distribution was assessed using flow cytometry. One representative experiment is shown, and statistical graphs are shown beneath. H represents the sub-G1 phase indicating the cell apoptosis rate. **P < 0.01

Cell cycle analysis of HNE-1 cells showed accumulation at G2 phase after treatment with 0.05 μmol/L evofosfamide under hypoxia compared with 5 μmol/L evofosfamide under normoxia. However, the sub-G1 phase was no greater than 2%, indicating that apoptosis did not occur in the treated cells (Fig. 2).

### Evofosfamide induces DNA damage

**Fig. 3:**
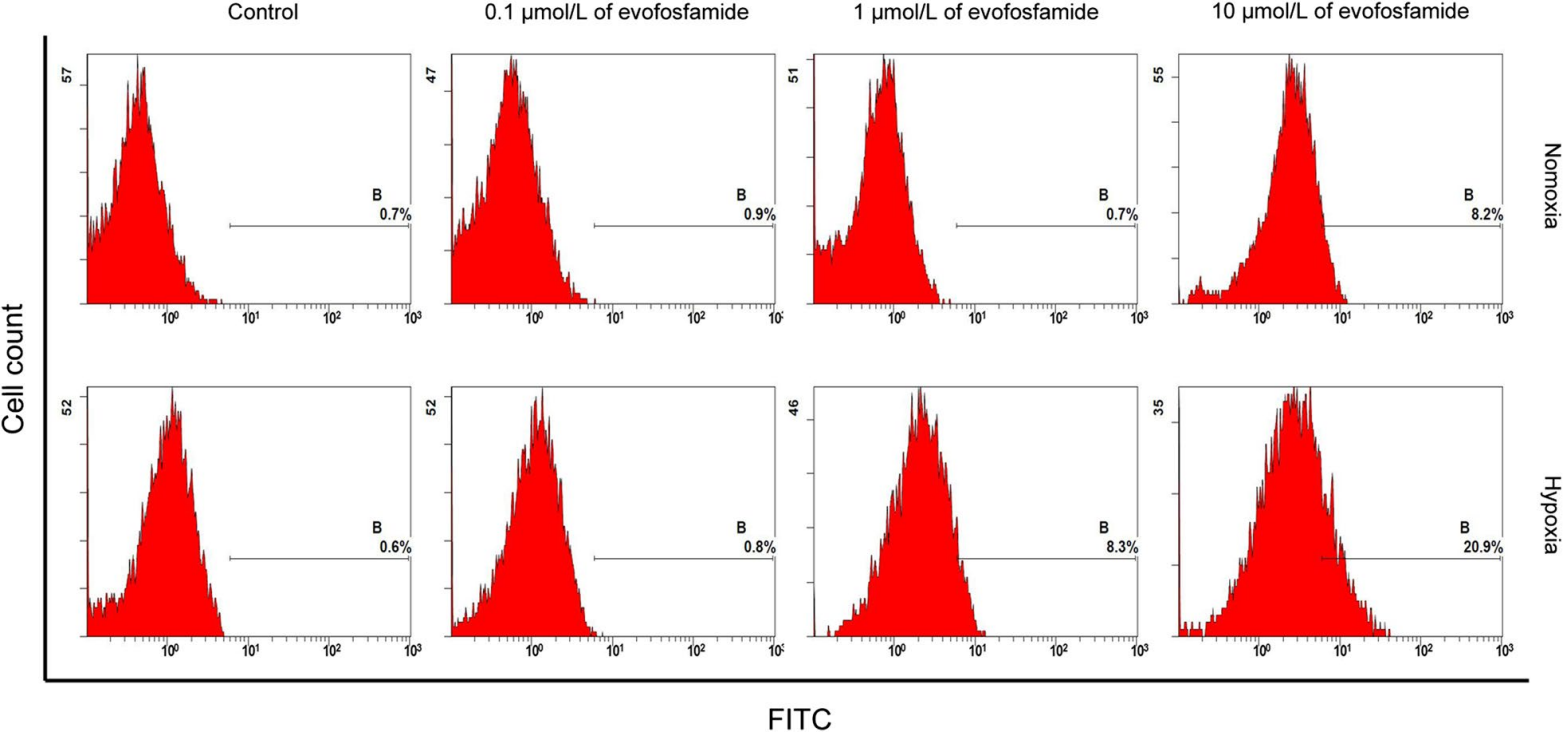
Assessment of DNA damage by flow cytometry. HNE-1 cells were cultured with evofosfamide for 24 h at the indicated concentration under hypoxia or normoxia

**Fig. 4:**
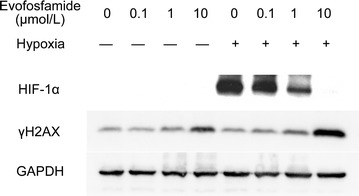
Western blot analysis of HIF-1α and γH2AX expression. HNE-1 cells were treated with evofosfamide for 24 h under hypoxia or normoxia and then harvested and lysed. Expression of HIF-1α and histone variant H2AX (γH2AX) was detected by western blot analysis with the indicated antibodies

Phosphorylation of the histone variant H2AX (γH2AX) in HNE-1 cells was examined by flow cytometry and western blot assays. After exposure to evofosfamide for 24 h, γH2AX expression increased to a greater extent under hypoxia than under normoxia (Figs. 3, 4).

### Evofosfamide decreases HIF-1α levels under hypoxic conditions

Cells were treated with increasing doses (0.1, 1, 10 μmol/L) of evofosfamide under normoxic and hypoxic conditions for 24 h. HIF-1α was induced under hypoxic conditions; however, this effect was decreased by treatment with evofosfamide. HIF-1α expression was almost completely suppressed at a high concentration (10 μmol/L) of evofosfamide (Fig. 4).

### Antitumor activity of evofosfamide in the NPC xenograft tumor model

**Fig. 5:**
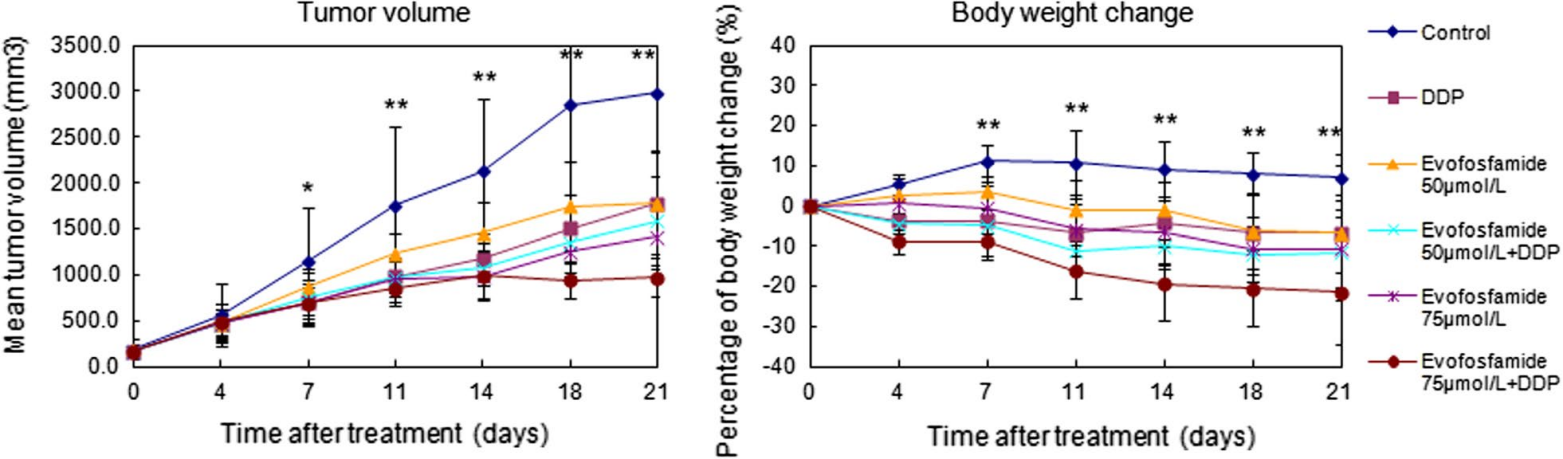
Antitumor activity of evofosfamide in the nasopharyngeal carcinoma (NPC) xenograft tumor model. Mice bearing HNE-1 NPC xenografts were treated with evofosfamide (50 or 75 mg/kg, intraperitoneally, twice a week for 2 weeks) and/or DDP (3 mg/kg, intraperitoneally, once a week for 2 weeks). The mean tumor volumes and percent body weight change for each group are shown. ^*^ *P *< 0.05, ^**^ *P *< 0.01

**Fig. 6:**
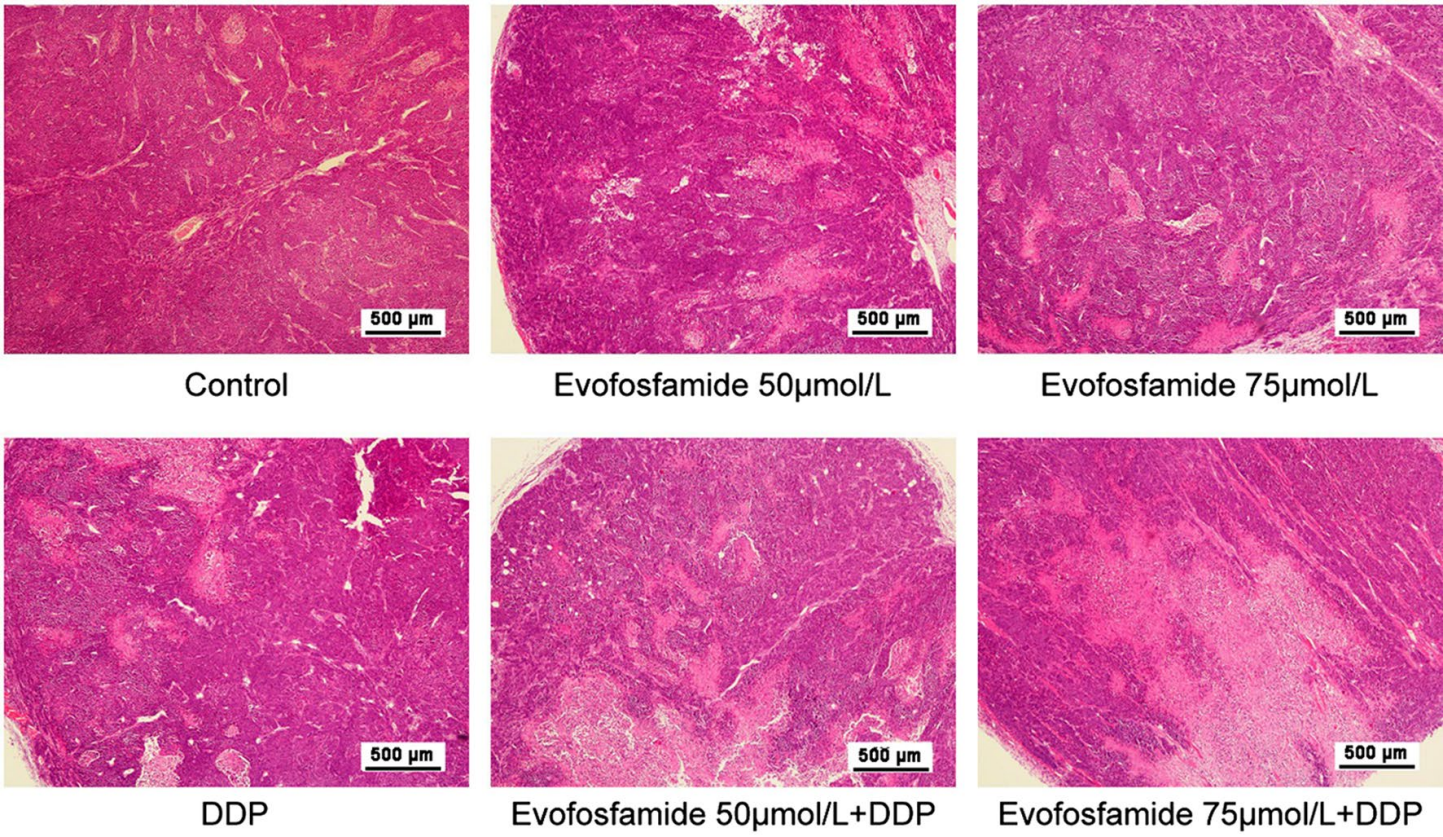
Tumor morphology by H&E staining. Tumor tissues were collected immediately after sacrifice, fixed in 4% paraformaldehyde, and embedded in paraffin. Hematoxylin and eosin (H&E) staining was conducted for tumor morphology. Representative images at 40× magnification are shown

We evaluated the efficacy of evofosfamide in the HNE-1 NPC xenograft model both as monotherapy and combined with DDP. The TGI values after administration of 50 and 75 mg/kg evofosfamide as a single agent twice a week for 2 weeks were 43% and 55%, respectively. Antitumor activity was also observed when evofosfamide was combined with DDP (3 mg/kg, q W × 2 W), with TGI values of 49% and 71%, respectively. However, the high-dose (75 mg/kg evofosfamide) combination resulted in severe body weight loss (≥ 20%). Antitumor activity of evofosfamide was also reflected by TGD. Tumor volume and body weight changes are presented in Fig. 5, and the TGI and TGD are shown in Table 3. Tumor morphology observed by H&E staining (Fig. 6) demonstrated necrotic fragments after treatment, as well as massive necrosis in the tumor tissue for the combined treatment groups.

**Table 3:** Tumor growth inhibition and tumor growth delay of evofosfamide in NPC xenograft models

Group	TGI (%)	TGD_500_ (day)	TGD_1000_ (day)
Control	0	10.4	13.2
DDP	42	11.4	18.3
Evofosfamide 50 μmol/L	43	11.2	15.4
Evofosfamide 50 μmol/L + DDP	49	11.3	18.9
Evofosfamide 75 μmol/L	55	11.4	21.2
Evofosfamide 75 μmol/L + DDP	71	11.2	N/A

TGD_500_ and TGD_1000_ were determined as the average increase in time for the treated tumor to reach a size of 500 or 1000 mm^3^ compared with the control group

*TGI* tumor growth inhibition, *TGD* tumor growth delay

### Evofosfamide reduces the hypoxic regions in tumor tissues

**Fig. 7:**
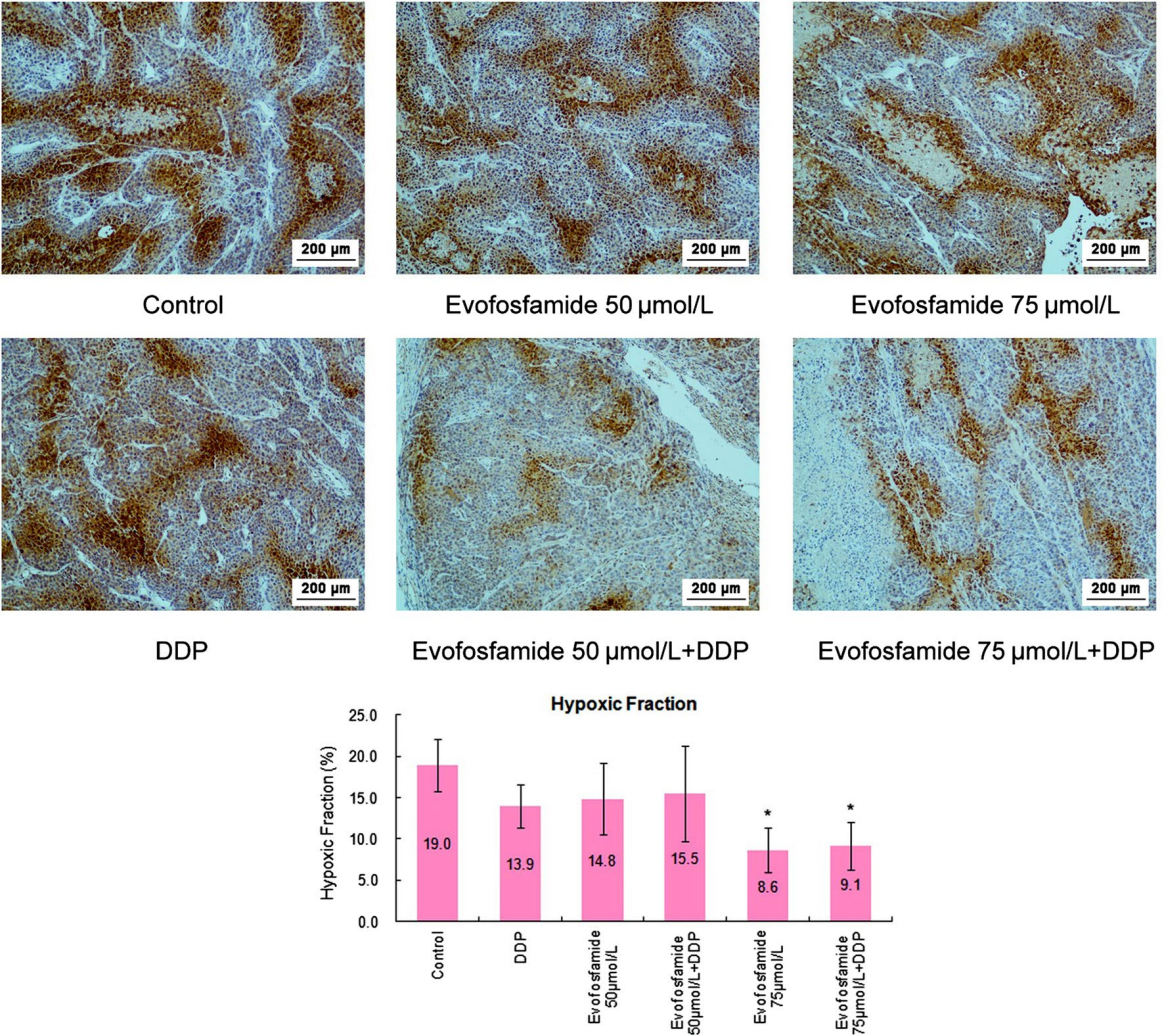
Detection of tumor hypoxic regions by immunohistochemical staining with pimonidazole. Pimonidazole-positive hypoxic areas in the whole tumor (hypoxic fraction) were extracted using Image-Pro Plus 6.0 for three animals in each group. Representative images at 100× magnification are shown. Statistical results are shown in the graph. *P < 0.05

The hypoxic regions in xenograft tumors were decreased by evofosfamide as both monotherapy and in combination treatment compared with the control group (Fig. 7). There was a statistically significant difference between the combined groups and control group (*P *< 0.05); however, there was no difference between the two different dose models.

## Discussion

Three poorly differentiated human NPC cell lines were examined in the current study. Both the cytotoxicity study and clone formation assay confirmed that evofosfamide exhibited hypoxia-selective cytotoxicity as a single agent in all three NPC cell lines. Additionally, the sensitization under hypoxia ranged from ninefold to greater than 300-fold. We also tested the effect of evofosfamide combined with DDP in these cell lines. Synergistic efficacy was observed after combination treatment of evofosfamide with DDP for 48 h, with a lower effective drug concentration under hypoxia than under normoxia.

The mechanisms underlying the hypoxia-selective cytotoxicity of evofosfamide were also explored in the present study. We showed significant G2-phase arrest after exposure to evofosfamide, especially under hypoxia, similar to previously reported findings for other solid tumors [13, 24]. Moreover, we did not observe apoptosis in the NPC cell lines after incubation with evofosfamide, in contrast to a study of evofosfamide in multiple myeloma [25]. Histone H2AX phosphorylation (γH2AX) is a robust and sensitive marker of DNA interstrand cross-linking [26]. DNA damage results in rapid phosphorylation of H2AX at Ser139 [22]. We showed that evofosfamide induced γH2AX in the NPC cell line HNE-1, indicating that evofosfamide induced DNA damage under hypoxic conditions. HIF-1α is an important reactive factor to hypoxia and its expression correlates with a poor prognosis in NPC [7, 8]. We found that HIF-1α was expressed under hypoxia and that its expression was suppressed by evofosfamide in a dose-dependent manner in HNE-1 cells, similar to findings in acute myeloid leukemia [21] and sarcoma [27]. The suppression of HIF-1α correlated with the reduction of hypoxic regions in vivo. However, how evofosfamide suppressed the expression of HIF-1α is not clear.

We also tested the antitumor activity of evofosfamide in NPC using human tumor xenograft models in immunocompromised mice. Evofosfamide treatment at two doses (50 and 75 mg/kg) showed antitumor activity both as a single agent and combined with DDP. Significant TGI was observed in all treatment models and hypoxic regions were reduced after evofosfamide treatment. However, there was no difference between the two doses employed. Considering body weight losses and agent efficacy, we concluded that 50 mg/kg evofosfamide is the optimal dose for nasopharyngeal carcinoma treatment.

It should be noted that we used only three cell lines in vitro and one cell line in vivo in this study, and different effects of evofosfamide might be observed in other cell lines. Additionally, drug toxicity and pharmacokinetics should be tested before clinical use.

## Conclusions

In conclusion, the results described here demonstrate that evofosfamide exhibits hypoxia-selective cytotoxicity in nasopharyngeal carcinoma and has synergistic efficacy when combined with DDP. The cytotoxicity appears to be related to cell cycle arrest, DNA damage, and suppression of hypoxia in a hypoxia-dependent manner. Our results provide rationale for the potential clinical application of evofosfamide for NPC.
